# Microbiome interplays in the gut-liver axis: implications for liver cancer pathogenesis and therapeutic insights

**DOI:** 10.3389/fcimb.2025.1467197

**Published:** 2025-01-28

**Authors:** Xuran Wang, Bin Zhang, Runqiu Jiang

**Affiliations:** ^1^ Medical School of Nanjing University, Nanjing, Jiangsu, China; ^2^ Department of Gastroenterology, Affiliated Nanjing Drum Tower Hospital, Medical School of Nanjing University, Nanjing, China; ^3^ Department of Clinical Laboratory, The First Affiliated Hospital of Anhui Medical University, Hefei, China

**Keywords:** HCC, gut-liver axis, gut microbiome, microbial metabolites, liver cancer

## Abstract

Globally, primary liver cancer (PLC) ranks the most fatal malignancy. Most of the patients are in advanced stage of PLC at the very time they are diagnosed with it, accounting much for its poor prognosis. With the advancement of modern medical research and care system, the main etiology of PLC more and more switches from hepatitis viruses such as HAV, HBV, HCV, HEV to other causes like metabolism-associated steatohepatitis (MASH) and metabolic-associated fatty liver disease (MAFLD). As a result, it is of great necessity to find out new ways for treatment and early diagnosis to cope with this problem. Nowadays, as the mechanism of the Gut-Liver Axis in the formation of MAFLD, MASH and PLC has been gradually elucidated. The association between gut microbiome and the formation of PLC is of great significance to take an insight into. In this review, we present the concept of Gut-Liver Axis and its function in the mutual influence between gut microbiota and PLC from several aspects in which we will focus on the structure of gut barrier and the functional influences the gut microbiota have on the immune response and metabolic changes on human liver. Furthermore, we conclude the potential association of gut microbiota constitution with the PLC. Eventually, we hope this review can offer novel instructions for early diagnosis and treatment for liver cancer.

## Introduction

PLC stands as the fourth leading cause of cancer-related mortality worldwide, marking the sixth most frequently diagnosed cancer and the third leading cause of cancer deaths in 2020, with an estimated 906,000 new cases and 830,000 fatalities (Feng et al., 2020).

PLC comprises three primary subtypes: hepatocellular carcinoma (HCC), intrahepatic cholangiocarcinoma (ICC), and combined HCC-ICC (cHCC-ICC). These subtypes exhibit distinct clinicopathological morphologies and genetic alterations. HCC is predominantly linked to chronic infection with hepatitis B virus (HBV) or hepatitis C virus (HCV), aflatoxin-contaminated foods, heavy alcohol intake, excess body weight, type 2 diabetes, and smoking. On the other hand, ICCs are associated with chronic liver inflammation and biliary tract diseases, while overweight, obesity, MASH, and liver cirrhosis collectively contribute to cHCC-ICC development (Feng et al., 2020).

Despite the understanding of nonviral risk factors such as alcohol abuse, metabolic syndrome, chronic liver inflammation, and biliary tract diseases, patients diagnosed with PLC are often in an advanced stage, leading to high mortality within 5 years. The absence of early diagnosis and prevention markers contributes to the low survival rate, emphasizing the urgent need for new detection methods and treatment options.

In recent years, studies on the gut-liver axis have provided novel insights into the pathogenesis of liver diseases, including PLC. The gut-liver axis represents the bidirectional communication between the gut microbiota, intestinal barrier, and liver. Gut microbiota significantly influence liver metabolism, immune responses, and inflammation through their metabolites, which include short-chain fatty acids (SCFAs) and bile acids (BAs). These metabolites play pivotal roles in modulating hepatic immune tolerance and maintaining metabolic homeostasis. Disruption of this axis has been implicated in various liver diseases, particularly MAFLD, MASH, and HCC (den Besten et al., 2013; Chun et al., 2019; Zhang et al., 2019).

Recent studies have elucidated the role of gut microbiota dysbiosis in the progression of liver diseases. For instance, it has been demonstrated that altered gut microbiota composition enhances the production of pro-inflammatory cytokines and metabolites that exacerbate liver inflammation, fibrosis, and tumorigenesis. Dysbiosis has been shown to increase levels of pathogenic bacteria such as Escherichia coli while reducing protective commensals like Firmicutes and Bifidobacterium (Wahlstrom et al., 2016).

The role of microbial metabolites has gained significant attention. BAs have been identified as crucial signaling molecules that modulate liver disease progression. Secondary BAs such as deoxycholic acid (DCA) can promote hepatocarcinogenesis by inducing chronic inflammation through the activation of inflammatory pathways like NF-κB. Conversely, beneficial metabolites such as SCFAs, particularly butyrate, can reduce inflammation and enhance gut barrier integrity (Gadaleta et al., 2011; Li et al., 2013; Collins and Patterson, 2020).

Additionally, recent advances in high-throughput sequencing and metabolomics have identified specific gut microbiota signatures associated with different stages of HCC. These findings have opened new avenues for non-invasive diagnostic markers and targeted therapies aimed at modulating the gut microbiota to prevent or treat liver cancer (Kim et al., 2018; Javdan et al., 2020; Brescia and Rescigno, 2021).

This review aims to provide a comprehensive analysis of the gut-liver axis and its multifaceted role in PLC. We explore the mechanisms linking gut microbiota and liver diseases, focusing on microbial metabolites, intestinal barrier integrity, and bile acid metabolism. Furthermore, we discuss therapeutic strategies targeting the gut-liver axis and propose future research directions to enhance our understanding of this intricate system.

## Controversies and challenges in current researches

Despite significant advances in understanding the gut-liver axis, several controversies remain unresolved, particularly concerning the causative role of gut dysbiosis in liver diseases.

Some studies (Sekirov et al., 2010) suggest that alterations in the gut microbiota directly contribute to liver inflammation and fibrosis through mechanisms such as increased intestinal permeability and endotoxin translocation while other studies (Zhang et al., 2019) argue that gut dysbiosis is a secondary phenomenon resulting from liver dysfunction, as evidenced by experiments where hepatic injury precedes microbial changes.

Another key area of contention lies in the role of microbial metabolites, such as SCFAs. While SCFAs are widely recognized for their anti-inflammatory properties, certain conditions—such as an imbalanced gut microbiota—may lead to excessive SCFA production, potentially exacerbating hepatic inflammation through immune cell activation. This dual role complicates the therapeutic targeting of SCFAs in liver diseases (den Besten et al., 2013; Chun et al., 2019).

In this review, we mainly focus on the metabolites which are produced and subsequently modified by the gut microbiota. We believe the gut microbiota, gut and liver are in a delicately dynamic balance in which they can influence and be influenced by each other.

By synthesizing current evidence and highlighting these controversies, we hope to provide a balanced perspective, offering insights into potential resolutions through interdisciplinary approaches and innovative research methodologies.

## Gut-liver axis

The gut-liver axis is a dynamic interplay involving three key components: the liver, intestine, and gut microbiota. These elements engage in intricate communication to regulate the homeostasis of the human body. Recent advancements have significantly enhanced our understanding of the sophisticated mechanisms governing these interactions. Subsequently, we will discuss each of these components and explore the communication and mechanisms under optimal physiological conditions.

### Liver

The liver plays a crucial role as a metabolic organ where several vital substances undergo complicated process, such as glucose, lipid and proteins. The metabolism of these substances involves more than just the liver, it comprises other parts like the gut microbiota and hormones secreted by other organs. Of note, the metabolism of BAs, Bilirubin is the bridge that connects both intestine, microbiota and liver. Additionally, the drug metabolism is also crucial. The metabolism progress is in such a dynamic balance, maintaining the homeostasis of human body.

The liver’s close anatomical relationship with the gastrointestinal (GI) system enables it to receive nutrients, hormones, and metabolites directly from the GI tract via the portal vein. Metabolites produced by gut microbiota are also transported to the liver through this route, where they play a crucial role in regulating the metabolism of glucose and fats. An imbalance in these metabolic processes can precipitate conditions such as insulin resistance, diabetes, and MAFLD.

In the enterohepatic circulation (EHC), BAs transport from liver to the intestine and finally return to the liver. This progress is finished by a variety of bacteria and requires amount of enzymes synthesized by liver. The synthesis and biotransformation of BAs will be discussed later. BAs are molecules synthesized in the liver from cholesterol, subsequently released into the gut where they undergo further metabolism by the microbiota (Wahlstrom et al., 2016). The quantity of BAs generated is subject to EHC, which involves communication between the gut and the liver. Prior to excretion, primary BAs undergo conjugation with the amino acids glycine and, to a lesser extent, taurine, after which they are released into the bile. After a meal, bile undergoes a reversal of direction in the terminal ileum, leading to the reabsorption of conjugated BAs through the gut epithelium. These BAs are recycled subsequently in the liver upon entering the portal circulation. Beyond their role in micelle formation and the absorption of fat and fat-soluble vitamins, BAs play an important role in shaping the microbiota. The crosstalk between BAs and the gut microbiota occurs at various points, highlighting the significance of this two-way interaction, where the microbiota influences bile acid metabolism, and in turn, BAs impact microbiota composition (Wahlstrom et al., 2016). Moreover, contrary to the initial belief that BAs merely undergo recirculation from the gut to the liver, recent findings emphasize that, upon transformation into secondary BAs they function as signaling molecules in the intestinal epithelium, primarily through the farnesoid X receptor (FXR).

Activation of FXR has been demonstrated to improve the properties of the epithelial barrier, facilitate the repair of GVB damage, and regulate the metabolic syndrome (Gadaleta et al., 2011). Conflicting outcomes have emerged from studies employing mice with a constitutive or epithelial-specific FXR knock-out, indicating distinct functions of FXR engagement in the development of MASH (Li et al., 2013). Mice lacking FXR exhibit resistance to diet-induced obesity, a phenomenon likely modulated by intestinal FXR and the microbiota (Li et al., 2013).

Recent studies have illuminated the ability of the gut microbiota to influence drug metabolism through both direct and indirect mechanisms (Kim et al., 2018; Collins and Patterson, 2020; Javdan et al., 2020). Directly, this is accomplished through the secretion of enzymes by the microbiota. Indirectly, microbial impact on the host occurs via their metabolites. In terms of direct functions, the microbiota can metabolize drugs through hydrolytic and reductive biotransformations. Furthermore, microbial metabolites may compete with drugs for host-metabolizing enzymes. Additionally, these metabolites can modulate immune cell dynamics during immunomodulatory interventions such as conditioning, and also alter the levels of drug metabolizing enzymes in the intestine and liver.

### Intestine

The intestine, a vital organ in the digestive system, comprises the small intestine and large intestine. The small intestine, consisting of the duodenum, jejunum, and ileum, serves as the primary site for digestion and nutrient absorption. Conversely, the large intestine, or colon, functions for absorption of water and electrolytes from food remnants, and also as a place accommodating diverse microbiota crucial for fermentation, vitamin synthesis, and gut health. Additionally, the large intestine forms and stores feces, aiding in waste elimination through peristaltic movements. Together, these intestinal functions ensure effective digestion, nutrient absorption, water balance maintenance, and waste excretion, supporting overall digestive and metabolic well-being.

Additionally, the ileum plays a prominent role in immune regulation, housing substantial lymphoid tissue such as Peyer’s patches, which contribute to immunological responses. Despite their collective involvement in the digestion and absorption of nutrients, each segment of the small intestine exhibits nuanced functional distinctions. According to one research which studies the amount of microbes in the human gut, the microbial density of small intestine microbiota is estimated at 10^3^ - 10^8^ cells/g, with an increasing gradient going from a low density in the duodenum (10^3^) to a high density in the terminal ileum (10^8^) which is still approximately 4-fold lower than in the colon (Sekirov et al., 2010).

The human intestine assumes a crucial responsibility in defending against harmful elements like microbes and their byproducts. Given the diverse functions of the intestine, a closer examination of its structure, particularly the intestinal barrier, becomes imperative. Fundamentally, the intestinal barrier comprises three key components: the mucus barrier, the epithelial barrier, and the gut-vascular barrier. Understanding these structural elements provides essential insights into the intricate workings of the intestine.

Of note, the gut barrier, also known as the intestinal barrier, is highly dynamic in a steady-state condition, and its upkeep is a collaborative endeavor involving specialized immune cells, epithelial cells, and accessory cells like glial cells and pericytes (Brescia and Rescigno, 2021). Any fluctuation in its structure will lead to immune reaction in its microenvironment and dysbiosis of gut microbiota, causing the translocation of microbes and their metabolites. The increased permeability of gut was previously seen as a result of dysbiosis of gut microbiota or the result of diseases. However, it is regarded as a causative agent of these conditions. It will lead to systemic dissemination of gut luminal contents and affect tissue function either directly or indirectly.

**Figure 1:**
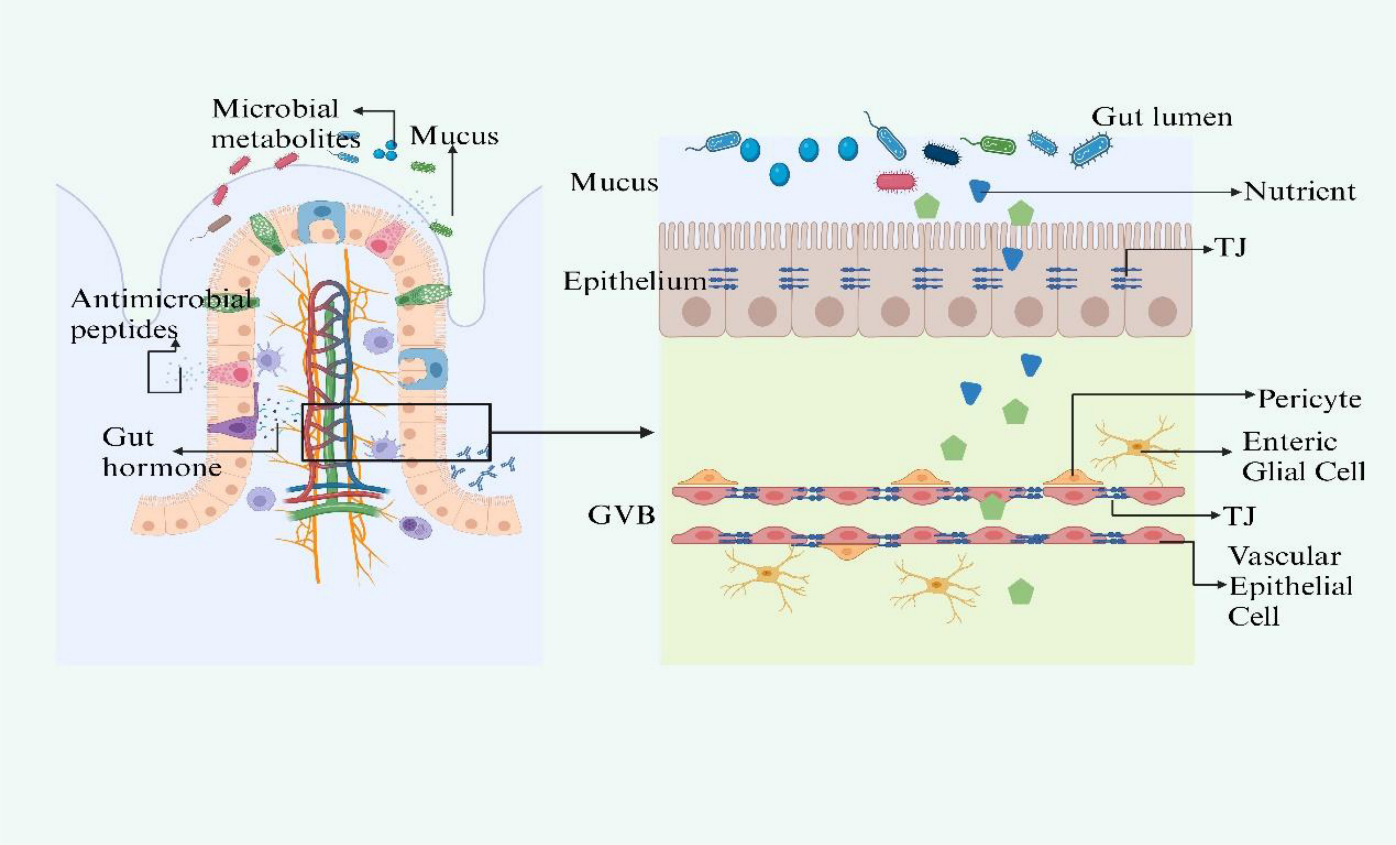
The structure of gut barrier. The intestinal barrier, comprising the mucus layer, epithelial barrier, and Gut Vascular Barrier (GVB), is essential for protection and interaction with the gut microbiota. Mucus secretion, regulated by ACh and VIP, protects epithelial cells and supports microbiota. Disturbances in mucus secretion can disrupt microbiota balance and the gut-liver axis. The epithelial barrier selectively allows nutrients and blocks harmful substances, with gut microbiota metabolites like SCFAs and indoles maintaining its integrity and modulating immunity. Specialized cells within the barrier use TLRs and NLRs to initiate immune defenses, while ILC3s, responsive to SCFAs and regulated by circadian rhythms, produce crucial cytokines like IL-17 and IL-22. The GVB, structurally akin to the BBB, is regulated by microbiota-influenced mechanisms that control molecule trafficking and intestinal health. Figure created with BioRender.com.

The intestinal barrier comprises three key layers: the mucus barrier, the intestinal epithelial barrier, and the Gut Vascular Barrier (GVB) (**Figure 1**). The mucus barrier, primarily formed of mucus and regulated by the enteric nervous system (ENS) is crucial for protecting epithelial cells from external substances and the gut microbiota (den Besten et al., 2013; Chun et al., 2019). The mucus barrier is the most associated layer with the gut microbiota due to their close distance. It provides a substrate and habitat for microbial colonization. Disruptions in mucus secretion may lead to microbiota imbalances and destabilize the gut-liver axis.

Beneath the mucus layer lies the intestinal epithelial barrier, a monolayer of tightly connected cells that selectively allows nutrient absorption and prevents the influx of pathogens (de Aguiar Vallim et al., 2013). The gut microbiota supports this barrier by generating metabolites such as short-chain fatty acids (SCFAs), indoles, and polyphenols, which preserve the epithelial turnover and integrity by modulating tight junction (TJ) gene expression (Rui, 2014). SCFAs also supply energy to colonocytes and modulate inflammation and immunity (Reynes et al., 2018; Zhang et al., 2021).

Specialized epithelial cells interspersed among enterocytes, including goblet and Paneth cells, detect bacterial molecules through Toll-like receptors (TLRs) and NOD-like receptors (NLRs), triggering immune responses (Wahlstrom et al., 2016). Group 3 innate lymphoid cells respond to microbiota via SCFA-sensitive receptors and involve in the production of IL-17 and IL-22, playing a vital role in the immune barrier (Gadaleta et al., 2011; Li et al., 2013; Collins and Patterson, 2020). The function of ILC3s is influenced by circadian rhythms and diet via VIP signaling (Kim et al., 2018; Javdan et al., 2020).

The GVB, comparable to the blood-brain barrier (BBB), is the last line of defense in the intestine, ensuring that microorganisms or toxins do not enter systemic circulation (Brescia and Rescigno, 2021). It has a unique composition, allowing for larger molecule diffusion, which is essential for inducing tolerance while preventing bacterial translocation (Bäckhed et al., 2005; Arumugam et al., 2011). The formation and maintenance of the GVB are influenced by the gut microbiota, which also impacts the development of the enteric glial cell network and intestinal angiogenesis (Wu et al., 2011). Any impairment of the three layers will lead to gut leakiness and result in the dysbiosis of gut microbiota. This disturbance disrupts the body’s homeostasis, paving the way for the pathogenesis of diseases, particularly those affecting the liver.

### Gut microbiota

At the heart of the gut-liver axis is the gut microbiota, an intricate community of microorganisms residing in the GI tract. The gut microbiota mostly consists of strict anaerobes, outnumbering facultative anaerobes and limited amount of aerobes. While over 50 bacterial phyla have been described, the human gut microbiota is mainly dominated by two: *Bacteroidetes* and *Firmicutes*. *Proteobacteria*, *Verrucomicrobia*, *Actinobacteria*, *Fusobacteria* and *Cyanobacteria* are present in smaller proportions (Bäckhed et al., 2005; Arumugam et al., 2011; Wu et al., 2011). Estimates of bacterial species in the human gut vary widely, ranging from approximately 500 to 1,000 species (Sekirov et al., 2010), highlighting the vast diversity within this ecosystem. The gut microbiota plays a crucial role in nutrient metabolism, immune modulation, and protection against pathogens. The gut microbiota is organically linked with host through some important substances such as the uptaken food and BAs with its specific enzyme systems.

The observation that germ-free (GF) animals require a significantly higher caloric intake to maintain the same body weight as specific pathogen-free (SPF) animals has led to investigations into how the gut microbiota maximizes caloric availability from ingested nutrients (den Besten et al., 2013). These mechanisms generally fall into two categories: extracting additional calories from otherwise indigestible oligosaccharides and promoting nutrient uptake and utilization by modulating the absorptive capacity of the intestinal epithelium and ultimate nutrient metabolism. During this process, the carbohydrate-active enzymes (CAZymes) of gut microbiota play a fundamental role in not only digesting these substances which cannot be absorbed by human beings, but also making gut microbiota better connect to the host body.

Numerous bacterial species are implicated in metabolizing dietary fiber to SCFAs, serving as a significant energy source for humans. Some SCFAs, like butyrate, not only provide energy but also prevent the accumulation of potentially toxic metabolic by-products, such as d-lactate (den Besten et al., 2013).

The gut microbiota functions a lot in the biotransformation of primary BAs to the secondary BAs. The gut microbiota interacts with primary BAs through various mechanisms. One key aspect is deconjugation of BAs, where the gut microbiota removes the taurine or glycine conjugates from primary BAs, converting them into their free forms. This process is primarily carried out by bile salt hydrolase (BSH)-producing bacteria (Sayin et al., 2013).

Furthermore, the gut microbiota plays a significant role in modulating the uptake and deposition of dietary lipids. It suppresses the inhibition of lipoprotein lipase, leading to increased LPL activity in adipose tissues and enhanced fatty acid uptake into adipocytes. Additionally, mono-association of germ-free mice with Bacteroides thetaiotaomicron upregulates the expression of colipase, a pancreatic lipase cofactor, promoting efficient hydrolysis of dietary lipids. Upregulation of a Na+/glucose cotransporter at the intestinal epithelium suggests increased glucose uptake. Gpr41, a G-protein-coupled receptor binding SCFAs, and peptide-YY (PYY), an enteroendocrine hormone, are involved in microbiota-dependent regulation of host energy balance (de Aguiar Vallim et al., 2013; Rui, 2014; Reynes et al., 2018).

## BAs as vital media of the connection of liver, gut and gut microbiota

Besides the anatomical proximity of the liver, gut and gut microbiota through portal vein which makes it easy to influence any of these components, leading to the diseases such as MAFLD, PLC, one metabolite makes them an organic entity, it’s the BAs.

**Figure 2:**
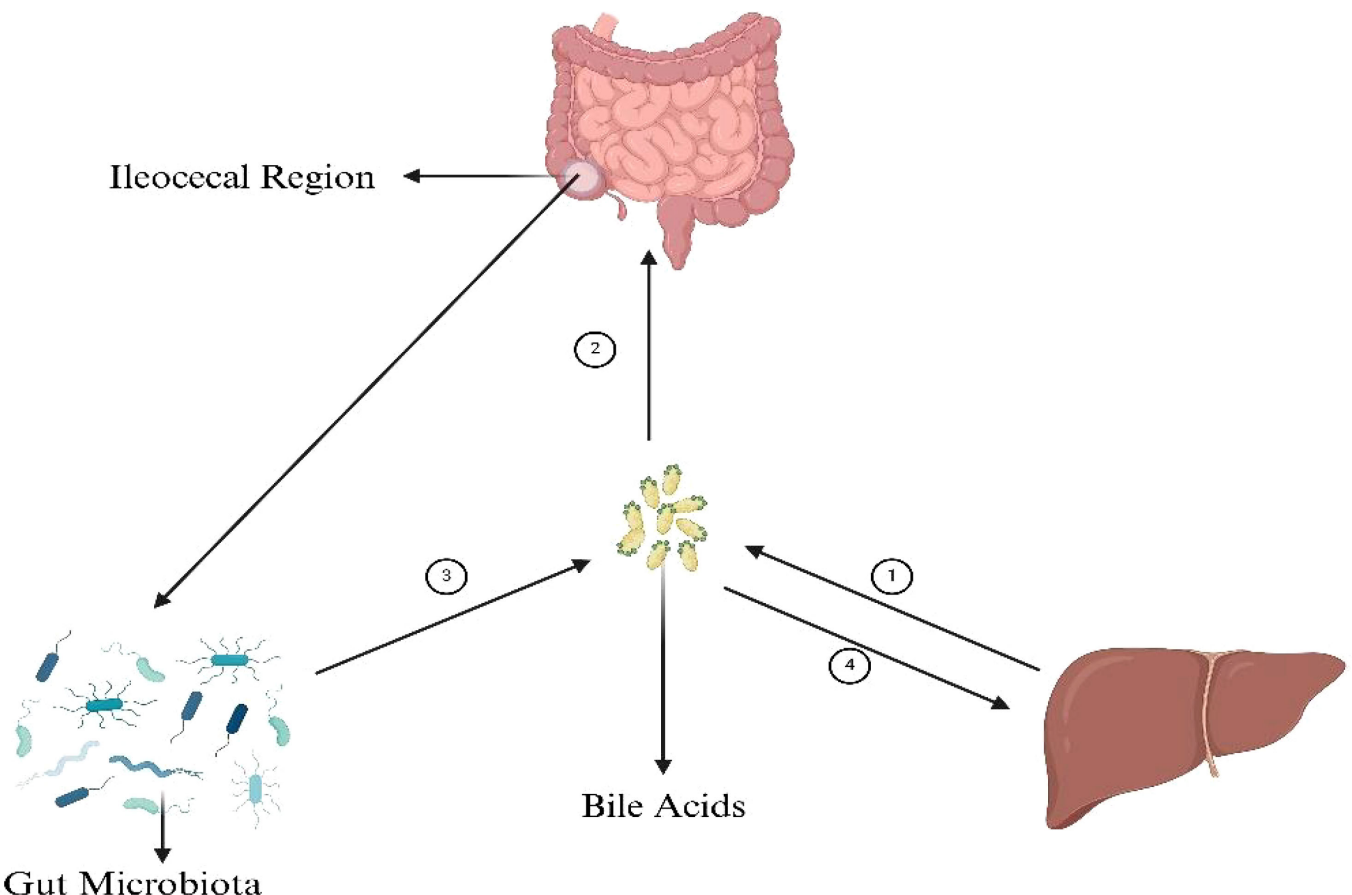
BAs’ connection with gut microbiota, liver and intestine. ① The synthesis of primary bile acids (BAs), cholic acid (CA) and chenodeoxycholic acid (CDCA), from cholesterol in the liver is a multifaceted process integral to lipid metabolism and cholesterol homeostasis. Initiated by the rate-limiting enzyme cholesterol 7α-hydroxylase (CYP7A1), cholesterol undergoes hydroxylation, followed by a series of modifications including further hydroxylations, epimerization, and bond breaking, leading to the formation of CA and CDCA. These compounds are then often conjugated with glycine or taurine to enhance solubility, resulting in the formation of glycocholic and taurocholic acid, and their CDCA equivalents. ② The absorption of bile acids in the ileum ensures the reuse of these acids in digestion and lipid regulation. BAs are actively transported from the intestinal lumen into enterocytes by the apical sodium-dependent bile acid transporter (ASBT). ③ The synthesis of secondary bile acids in the ileum by gut microbiota involves crucial steps: deconjugation of primary bile acids by the enzyme bile salt hydrolase (BSH), and the key transformation through 7α-dehydroxylation, primarily performed by specific anaerobic bacteria like Clostridium and Eubacterium, converting cholic acid to deoxycholic acid (DCA) and chenodeoxycholic acid to lithocholic acid (LCA). These processes, facilitated by gut microbiota, play a significant role in diversifying the bile acid pool and impacting digestive and metabolic health. ④ Secondary bile acids, after being transported back to the liver through the enterohepatic circulation, play significant roles in metabolic regulation primarily through interacting with key receptors such as the farnesoid X receptor (FXR) and the G protein-coupled bile acid receptor (TGR5). These interactions influence cholesterol metabolism, glucose homeostasis, lipid metabolism, and energy expenditure, highlighting the critical role of secondary bile acids in maintaining metabolic health and signaling pathways. Figure created with BioRender.com.

**Figure 3:**
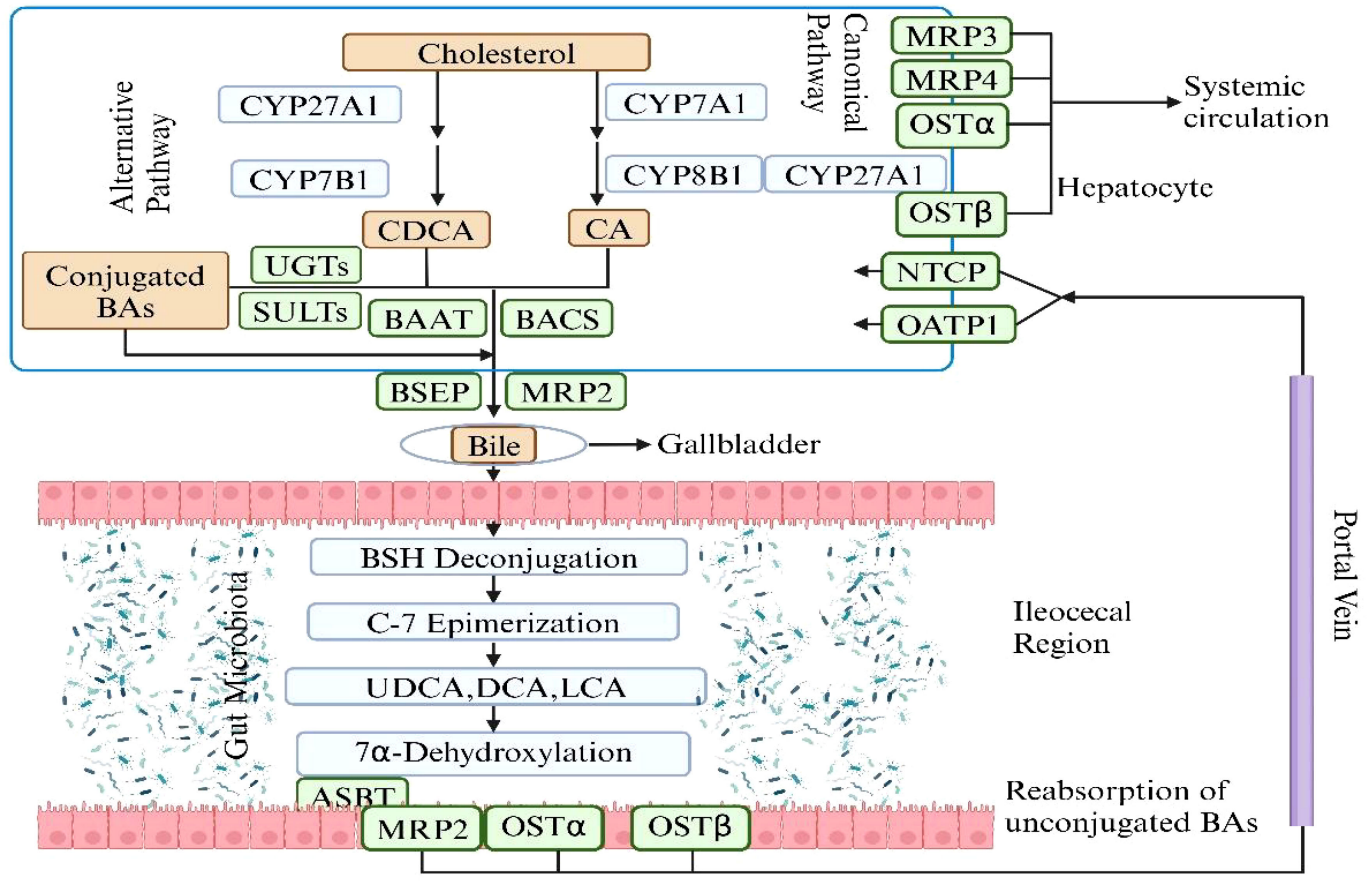
The synthesis of Primary BAs and Secondary BAs. The metabolic pathway of bile acids begins with the conversion of cholesterol in the liver into primary bile acids—cholic acid and chenodeoxycholic acid—via the enzyme cholesterol 7α-hydroxylase and other enzymatic reactions. These acids are subsequently conjugated with glycine or taurine to enhance solubility and functionality, and are then secreted into the intestine via bile. In the intestine, gut bacteria metabolize these primary bile acids into secondary bile acids such as deoxycholic and lithocholic acids, which are absorbed back into the bloodstream and returned to the liver for reuse through deconjugation, epimerization. This cyclic process, known as enterohepatic circulation, is essential for effective lipid digestion, cholesterol regulation, and liver function maintenance. Figure created with BioRender.com.

The enterohepatic BAs axis serves as a vital link among the liver, intestine, and gut microbiota, orchestrating a sophisticated array of metabolic and signaling pathways. Primary BAs, synthesized in the liver from cholesterol, are secreted into the intestine, playing a pivotal role in lipid digestion. Their reabsorption in the ileum via transporters such as the apical sodium-dependent bile acid transporter (ASBT) is a testament to the efficiency of the enterohepatic circulation. A fraction of these BAs, however, eludes reabsorption and is subject to microbial biotransformation by gut microbiota, notably by species like Clostridium and Eubacterium (Sayin et al., 2013). These organisms employ enzymes, including BSH, to deconjugate primary BAs, subsequently transforming them through 7α-dehydroxylation. The secondary BAs produced are reabsorbed and engage hepatic receptors like the farnesoid X receptor (FXR) and the G protein-coupled bile acid receptor (TGR5), modulating hepatic metabolic functions and systemic energy balance. In response, the liver adjusts bile acid synthesis, creating a regulatory feedback loop that controls the size and composition of the bile acid pool and cholesterol metabolism (Zhang et al., 2021). This process underscores the enterohepatic BAs axis’s essential contribution to nutrient metabolism and its extensive impact on metabolic regulation (**Figures 2**, **3**).

The gut microbiota is essential in the synthesis of secondary BAs and plays a significant indirect role in regulating homeostasis via hepatic receptors, which is crucial for host metabolism and health. Primary BAs, once secreted into the intestine, undergo deconjugation by microbial bile salt hydrolases. These deconjugated BAs are then transformed by gut bacteria through processes like 7α-dehydroxylation into secondary BAs such as deoxycholic acid (DCA) and lithocholic acid (LCA). These secondary BAs are potent ligands for the FXR in the liver, regulating BAs synthesis by inhibiting the enzyme CYP7A1 and influencing lipid and glucose metabolism. They also interact with TGR5, which impacts energy balance, glucose regulation, and inflammatory responses. The metabolic modulation by secondary BAs via these hepatic receptors contributes to cholesterol homeostasis and insulin sensitivity, offering protection against metabolic diseases. Additionally, their immunomodulatory effects are pivotal in managing liver inflammation and disease. This interplay between secondary BAs and liver receptors through the gut microbiota also emphasizes the gut-liver axis’s critical role to maintain systemic health (Priyadarshini et al., 2018; Chun et al., 2019; Yin et al., 2023). The involvement of gut microbiota in this process highlights its fundamental role in physiological regulation beyond the gastrointestinal tract.

## Gut-liver axis in pathogenesis of liver disease

As is described in the context before, the 3 key components of gut-liver axis communicate with each other and are in a status of dynamic balance, co-contributing to the homeostasis of human body. As a result, once one or several of them dysfunctions, diseases may occur. Of the many results which lead to the dysfunction of these 3 components, the changes occur to the metabolism of BAs are the key of the pathogenesis. In the following content, we will focus on the alterations of BAs metabolism and the mechanisms behind it.

The gut barrier is the most vital defense of the stability of gut-liver axis. SCFAs are important ingredients in maintaining the integrity of gut barrier. *In vitro* studies, SCFAs have been proved to stimulate subepithelial myofibroblasts, leading to the production of prostaglandin, which in turn triggers mucin expression by epithelial cells (Willemsen et al., 2003). Butyrate specifically has been found to directly induce mucin production in human polarized goblet cell lines and in perfused rat colon (Gaudier et al., 2004). Additionally, SCFAs can enhance the production of antimicrobial peptides, such as the cathelicidin LL37 (Jiang et al., 2013), in human epithelial cell lines. The activation of GPR43 by butyrate has been observed to promote the production of β-defensins and RegIIIγ in both human and mouse colonic cell lines, through the stimulation of mTOR and STAT3 phosphorylation. The epithelial barrier restricts the entry of harmful insults originating from the external environment or the commensal microbiota (Odenwald and Turner, 2017). Conversely, the gut microbiota plays a pivotal role in preserving the physiology of the epithelial barrier by producing various metabolites, including SCFAs, indoles, and polyphenol metabolites. These metabolites contribute to the maintenance of epithelial cell turnover and barrier integrity by regulating the expression of TJ genes (Ghosh et al., 2021). Moreover, SCFAs serve as a primary energy source for colonocytes and exhibit a range of anti-inflammatory properties. They influence mucosal immune cell migration, cytolytic activity, cytokine production, and the secretion of antimicrobial peptides by intestinal epithelial cells and macrophages (Zhao et al., 2018; Schulthess et al., 2019). Furthermore, contrary to conventional thoughts that the intake of soluble fibers can do good for health, one recent study shows that the unregulated fermentation of fibers by gut microbes will lead to cholestatic liver cancer (Singh et al., 2018). Emerging evidence point to the potential role of gut microbiota in the pathogenesis of various liver diseases.

The gut-liver axis maintains health through complex interactions among the liver, intestine, and gut microbiota. BAs and microbial metabolites have great impact on liver function and gut barrier integrity. Disruptions in BA metabolism and gut barrier function is closely related to liver diseases. In the next section, we will explore how the composition of intestinal microbiota differs in patients with HCC compared to healthy individuals.

## Composition of intestinal microbiota in patients with HCC varies and is different from healthy people

The role of gut microbiota in the development and progression of liver diseases is considerably more significant than initially considered. Through a complex system of interactions within the gut-liver axis, disruptions in gut homeostasis may specifically trigger an inflammatory response within the liver and subsequent fibrogenesis. The increased production of pro-inflammatory cytokines by the host immune system largely depends on the presence of microbial products such as lipopolysaccharides (LPS) or other pathogen-associated molecular patterns (PAMPs) or microbe-associated molecular patterns (MAMPs). These can be recognized by the pattern recognition receptors (PRRs) expressed on the surface of gut cells or cells in portal circulation, initiating cascades of immune responses in the liver. Consequently, alterations in gut microbes contribute to various liver diseases, and in some cases, different cancers. According to several recent studies, the profile of gut microbiota in patients with liver diseases such as cirrhosis, MASH, MAFLD, HCC, and iCCA differs (Grat et al., 2016; Ponziani et al., 2019; Rodriguez-Diaz et al., 2022). Moreover, the degree of liver insufficiency is closely related to the severity of gut dysbiosis. One study conducted in 15 patients with HCC and 15 non-HCC patients shows that the presence of HCC was associated with a significant increase in fecal counts of *Escherichia coli* (Grat et al., 2016). In another study, researchers highlighted the association of the degree of gut microbial dysbiosis with the progression of HCC, and they observed that with the development of HCC, the dysbiosis tended to increase as well. Despite the significant difference in alpha diversity of the microbiota between the advanced stages of HCC (stage III and IV primary HCC)and healthy controls, the variation between early HCC and healthy controls was not as pronounced (Ni et al., 2019). However, the diversity of microbes in patients with HCC increases compared to patients with liver cirrhosis. Moreover, while the relative abundances of *Firmicutes* were not significantly changed in patients with primary HCC, Proteobacteria were significantly increased in patients with stages II and III primary HCC compared to healthy controls (Ni et al., 2019). It is noteworthy that most pro-inflammatory bacteria come from *Proteobacteria*, while many probiotic bacteria come from *Firmicutes* (Stecher, 2013). This result implies that pro-inflammatory bacteria accompany the development of primary HCC. Simultaneously, many pro-inflammatory bacteria in *Proteobacteria*, such as those of *Enterobacteriaceae*, serve as indicators of dysbiosis. High-quality sequencing found that compared with healthy controls, *Actinomyces, Atopobium, Desulfococcus, Enterobacter, Paraprevotella, Planctomycetes, Prevotella, Veillonella*, and many unidentified genera were enhanced in patients with stage I HCC. *Desulfococcus, Enterobacter, Lactococcus, Leptotrichia, Paraprevotella, Planctomycetes, Prevotella, Veillonella*, and many unidentified genera were enriched in patients with stage II HCC. *Actinomyces, Atopobium, Desulfococcus, Enterobacter, Haemophilus, Lactococcus, Leptotrichia, Neisseria, Oribacterium, Prevotella, Rothia, Selenomonas, Veillonella*, and many unidentified genera were multiplied in patients with stage III HCC. *Desulfococcus, Enterobacter, Prevotella, Veillonella*, and many unidentified genera were increased in all stages of HCC. However, *Acidaminococcus, Cetobacterium, Coprobacillus, Pyramidobacter, Turicibacter*, and two unidentified genera were reduced in patients with stage I HCC; and *Anaerotruncus, Cetobacterium*, and an unidentified genus were decreased in patients with stage II HCC. Moreover, *Acidaminococcus, Anaerostipes, Anaerotruncus, Butyricimonas, Cetobacterium, Cloacibacillus, Coprobacillus, Holdemania, Methanobrevibacter, Odoribacter, Pyramidobacter, Turicibacter*, and four unidentified genera were reduced in patients with stage III HCC. *Cetobacterium* was reduced in all stages of primary HCC. When it comes to the alterations of gut microbiota profile in HCC patients compared to normal people, one study points out that the Proteobacteria is increased in HCC patients with an simultaneous increase of dysbiosis (Ni et al., 2019). Several other researches revealed the fact that people with HCC do have a different profile of gut microbiota from that of normal people and the main variation lies in the increase of *Escherichia coli, Neisseria, Klebsiella* etc. and the decrease of *Firmicutes, Clostridium and Bifidobacterium* etc (Ni et al., 2019; Ren et al., 2019; Zhang et al., 2019; Zheng et al., 2020). In addition to these alterations in gut microbiota, there are other changes reported in different studies, and they will all be summarized in **Table 1** (Ponziani et al., 2019; Zhang et al., 2019; Liu et al., 2018; Zheng et al., 2020; Ren et al., 2019; Behary et al., 2021).

**Table 1:** Alterations of microbiota in HCC patients compared with normal people.

Levels	Alterations of gut microbiota	References
Phylum	*Proteobacteria ↑, Firmicutes ↓, Bacteroidetes ↓*	(Ni et al., 2019; Zhang et al., 2019)
Class	*Gammaproteobacteria ↑, Bacilli ↑, Clostridia ↓, Erysipelotrichia ↓*	(Zhang et al., 2019; Behary et al., 2021)
Order	*Lactobacillales ↑, Oscillospirales ↓*	(Zhang et al., 2019; Behary et al., 2021)
Family	*Enterobacteriaceae ↑, Enterococcaceae ↑, Oscillospiraceae ↓*, *Erysipelotrichaceae ↓*	(Zheng et al., 2020; Behary et al., 2021)
Genus	*Enterobacter ↑, Desulfococcus ↑, Prevotella ↑, Veillonella ↑, Cetobacterium ↓, Akkermansia ↓, Bifidobacterium ↓, Neisseria ↑, Limnobacter ↑, Phyllobacterium ↓, Clostridium ↓, Ruminococcus ↓, Coprococcus ↓, Klebsiella ↑, Haemophilus ↑, Alistipes ↓, Phascolarctobacterium ↓, Subdoligranulum ↓*	(Ni et al., 2019; Ponziani et al., 2019; Ren et al., 2019; Zheng et al., 2020)
Species	*Escherichia coli ↑, Enterobacter ludwigii ↑, Bacteroides xylanisolvens ↑, Bacteroides caecimuris ↑, Ruminococcus gnavus ↑, Clostridium bolteae ↑, Veillonella parvula ↑*	(Grat et al., 2016; Zhang et al., 2019; Behary et al., 2021)

The patients with HCC show variations in their gut microbiota. With the progression of the cancer, dysbiosis of gut microbiota occurs. Characterized by the increases of some bacteria like *Proteobacteria*, *Enterobacter Bacteroides* and decreases of *Cetobacterium*, *Akkermansia*, *Bifidobacterium* and *Clostridium*.

↑ means increase and the ↓ means decrease.

Of note, one recent study points out that the enhanced mitochondria activity reshapes a gut microbiota profile and puts off the progression of MASH (Juarez-Fernandez et al., 2023). According to the research findings, the absence of methylation-controlled J protein (MCJ), which acts as an inhibitor of mitochondrial complex I, has been shown to reduce hepatic injury and improve the gut-liver axis in a highly aggressive MASH dietary model (Juarez-Fernandez et al., 2023). This suggests that the enhanced mitochondrial activity is associated with changes in the gut microbiota profile, exerting protective effects through the enhancement of short-chain fatty acids, nicotinamide adenine dinucleotide (NAD+) metabolism, and sirtuin activity. Mitochondrial dysfunction, oxidative stress, and subsequent alterations in the gut microbiota collectively contribute to the development of MASH and even HCC (Aragones et al., 2019; Ni et al., 2019; Hsu and Schnabl, 2023; Juarez-Fernandez et al., 2023). Dysfunction of the mitochondria leads to increased production of reactive oxygen species (ROS), which can impact the composition of the gut microbiota by modulating the integrity of the intestinal barrier and triggering an immune response. MCJ-knockout (MCJ-KO) mice demonstrated an improved status of the gut-liver axis, along with downregulation of hepatic TLR-4 and NLRP3. Furthermore, MCJ-KO mice exhibited a distinct gut microbiota composition independent of the diet, characterized by an increase in the *Dorea genus* and a reduction in *AF12, Allobaculum*, and *Ruminococcus* (Chu et al., 2018).

Intestinal microbiota in HCC patients shows distinct differences from healthy individuals. Patients with HCC have increased levels of pro-inflammatory bacteria like *Escherichia coli* from the *Proteobacteria* phylum and decreased levels of beneficial *Firmicutes* such as *Clostridium* and *Bifidobacterium*. This imbalance in gut microbiota correlates with HCC progression. Additionally, mitochondrial activity influences gut microbiota composition, suggesting that mitochondrial dysfunction can impact microbial profiles and liver disease. The following section will delve into how changes in gut microbiota contribute to PLC, exploring the mechanisms through which these microbial shifts promote liver pathology.

## Mechanisms by which gut microbiota promotes PLC

The compromised integrity of the gut, resulting from damage to the gut barrier components (mucus barrier, intestinal epithelial barrier, and GVB), along with bacterial dysbiosis, MAMPs, bacterial metabolites, and the potential co-metabolism between microbes and the host, constitutes pivotal pathways driving liver inflammation, fibrosis, and genotoxicity that contribute to cancer promotion. In this part, our focus will be on elucidating the collaborative impact of pathogen-associated molecular patterns (PAMPs)/MAMPs-PRR axis and the metabolites of the gut microbiota on instigating immune responses and inflammations that foster the genesis of HCC.

### PAMPs/MAMPs-PRRs axis

Intestinal dysbiosis is concomitant with the loss of gut barrier integrity, facilitating the transfer of PAMPs to the portal circulation (Mouries et al., 2019). This process induces the activation of PRRs such as TLRs and NLRs in liver cells. Subsequently, these activations initiate pro-inflammatory signaling cascades, culminating in local inflammatory responses within the liver (Arab et al., 2018). TLRs represent a category of PRRs that typically experience suppression under normal, healthy liver conditions (Miura and Ohnishi, 2014). The delivery of pathogens and the molecules synthesized by them to the liver triggers the activation of TLR signaling. This, in turn, results in an upregulation in the production of cytokines such as Tumor Necrosis Factor α (TNF-α) and Interleukin-1β (IL-1β), both known for their actions against bacteria and viruses. Prolonged stimulation leading to elevated TLR signaling and increased expression of these cytokines can exacerbate hepatic injury in various liver diseases (Kiziltas, 2016). The associated MAMPs and PRRs with their associated diseases are summarized in **Table 2**. For instance, MASH is recognized for impacting the levels of TLR2, TLR4, TLR5, and TLR9. These receptors are activated by microbial antigens like LPS, peptidoglycan, flagellin and bacterial DNA etc., setting off inflammatory signaling cascades. Elevated systemic levels of LPS, a component of Gram-negative bacteria, are observed in liver diseases such as MAFLD and MASH. Injecting LPS into a mouse model for MAFLD enhances liver injury and increases the expression of pro-inflammatory cytokines (Jones et al., 2021). Wild-type mice fed a high-fat diet develop steatohepatitis with increased TLR4 expression and pro-inflammatory cytokines (Dapito et al., 2012). Furthermore, TLR4 mutants show resistance to LPS-induced release of pro-inflammatory cytokines, confirming the role of TLR4 signaling in MAFLD and MASH (Dapito et al., 2012). The presence of bacterial DNA, higher in MASH patients, leads to increased expression of TLR9 in MASH models (McGlasson et al., 2017). Experiments with TLR9-deficient models fed a choline-deficient amino acid-defined (CDAA) diet show less inflammation, steatosis, or fibrosis compared to wild-type models (Garcia-Martinez et al., 2016). TLR9 signaling influences the expression of inflammasome in macrophages, resulting in the formation of proinflammatory IL-1β and enhancing the progression of hepatic injury in MASH. TLR2 interacts with Gram-positive bacterial cell wall components like lipoteichoic acids and peptidoglycan. Based on experimental observations in mice models, insulin resistance induced by a high-fat diet can be prevented by inhibiting TLR2 signaling (Himes and Smith, 2010). Moreover, *TLR2*-knockout mice exhibit resistance to CDAA-induced steatohepatitis, accompanied by a decrease in the expression of proinflammatory cytokines (Miura et al., 2013). In contrast, TLR2-deficient mice on a ‘Methionine-Choline Deficient’ (MCD) diet exhibit comparable or even more severe steatohepatitis compared to wild-type mice. While the MCD diet may induce features of steatohepatitis, it contributes to increased insulin sensitivity and promotes weight reduction. Conversely, high-fat and CDAA diets result in weight gain and insulin resistance (Van Herck et al., 2017). TLR5 binds to bacterial flagellin and plays a protective role for the intestine. *TLR5*-knockout mice not only develop obesity and steatosis but also exhibit an imbalance in the gut microbiome. Moreover, the transfer of gut microbial communities from TLR5 knockout mice to wild-type (WT) germ-free mice results in the onset of metabolic syndrome. Thus, an interplay between the gut microbiome and TLR5 likely contributes to the pathophysiology of metabolic syndrome. Previous studies have also demonstrated elevated circulating levels of LPS in mice and patients with Chronic Liver Disease (CLD) as well as in HCC due to the presence of a leaky gut during various stages of CLD and hepatocarcinogenesis (Yu et al., 2010). Functional experiments conducted in germ-free, gut-sterilized, TLR-deficient, and LPS-treated mice have provided evidence that the leaky gut, facilitated by LPS and its receptor TLR4, makes essential contributions to hepatocarcinogenesis. TLR4 is present in multiple hepatic cell types, including Kupffer cells, hepatic stellate cells (HSCs), endothelial cells, and hepatocytes. LPS from the leaky gut appears to promote hepatocarcinogenesis via multiple cellular targets, including HSCs, the hepatocyte–tumor compartment, as well as liver-resident Kupffer cells. In HSCs, TLR4 activation leads to an NF-κB-mediated upregulation of the hepatomitogen epiregulin (Dapito et al., 2012). Another crucial mechanism through which the LPS–TLR4 axis promotes HCC formation is NF-κB-mediated prevention of hepatocyte apoptosis. Consequently, the expression of the apoptosis marker cleaved caspase 3 in TLR4-deficient and gut-sterilized mice is inversely correlated with the formation of tumors. Moreover, it has been demonstrated that the activation of the LPS–TLR4 signaling pathway in Kupffer cells leads to TNF-α and IL-6 dependent compensatory hepatocyte proliferation, as well as reduced oxidative stress and apoptosis (Yu et al., 2010). In addition, TLR4 activation in HCC cell lines by LPS enhances their invasive potential and induces the epithelial–mesenchymal transition (Jing et al., 2012; Yu and Schwabe, 2017).

**Table 2:** MAMPs with their PRRS and associated diseases.

MAMPs	PRRs	Associated diseases
LTA, Peptidoglycan	TLR2	MASH, Insulin Resistance
LPS	TLR4	MASH, MAFLD
Bacterial Flagellin	TLR5	Obesity, MASH
Bacterial DNA	TLR9	MASH

MAMPs like LTA, peptidoglycan are in association with TLR2 and will lead to diseases like MASH and insulin resistance. LPS with TLR4 can cause MASH and MAFLD. Bacterial Flagellin with TLR5 may trigger obesity and MASH. Bacterial DNA can also result in MASH.

The leaky gut is also a vital part in the MAMP-PRR axis. As is mentioned before, many cells that are interspersed among the enterocytes and highly specialized such as goblet cells, Paneth cells, neuroendocrine cells, tuft cells, and M cells, all express TLRs and NLRs. Once they identify the MAMPs released by the microbes, defense mechanisms will be triggered and proinflammatory cytokines like TNF and IL will be produced by immune cells. With the increased permeability of gut, these cytokines may well find their way to liver through GVB and portal circulation, resulting in inflammation and even cirrhosis in the liver.

## Mechanisms and effects of microbial metabolites on liver

The gut microbiota exists in a commensal relationship with our body. Under healthy conditions, microbial products, or the so-called microbial metabolites help maintain the homeostasis of our body. SCFAs is a cluster constituted by fatty acids like butyrate, propionate and acetate, etc. SCFAs not only contribute to the integrity of the gut but also function as essential components in vital signaling pathways. SCFAs are formed in the large intestine as a result of dietary assimilation of polysaccharides, resistant starch, fiber, etc (Clifton, 2001). The SCFAs work as nutrient and energy source for intestinal epithelium and act as precursors for lipogenesis and gluconeogenesis (den Besten et al., 2013). The butyrate level in the gut helps in maintaining the intestinal integrity as well as permeability (Silva et al., 2020). SCFAs bind and activate G-protein coupled receptors (GPCRs) GPR41 and GPR43 (Priyadarshini et al., 2018). This activation influences PYY secretion as well as causes inhibition of gut motility, thereby increasing the nutrient utilization and yielding of energy. The signaling across GPR41 and GPR43 leads to secretion of GLP1 which in turn reduces the food intake as well as emptying of gastric tract (Priyadarshini et al., 2018). Further, GPCR signaling also affects regulation of fatty acid oxidation and insulin sensitivity by hepatocytes. Apart from this, GPR43 activation also leads to inhibition of lipolysis and reduced plasma fatty acids (Priyadarshini et al., 2018). In addition to GPCR-based signaling, SCFAs can reach the liver through the portal circulation and can have either beneficial or deleterious effects on the liver. For example, increased acetate can be channeled to fatty acid biosynthesis pathway, thereby leading to triglyceride accumulation which has often been correlated to liver ailments (den Besten et al., 2013) (Aragones et al., 2019) Similarly, propionate which acts as a precursor for gluconeogenesis has also been associated to MAFLD (den Besten et al., 2013; Jasirwan et al., 2019) On the other hand, butyrate may utilize multiple mechanisms to reduce the pathophysiology associated with liver diseases. For instance, butyrate can activate AMP activated Protein Kinase (AMPK), which in turn reduces inflammation and influences glucose as well as lipid metabolism. AMPK further suppresses lipogenic genes (den Besten et al., 2013). AMPK expression in liver (regulated by butyrate) reduces insulin resistance and obesity. Butyrate can also function as inhibitors of “ Histone deacetylases” (HDACs) which can prevent development of liver diseases like MASH and MAFLD at epigenetic level (den Besten et al., 2013). However, in the presence of dysbiosis in the gut microbiota, alterations in microbial metabolites can pose a significant threat to our body.

**Figure 4:**
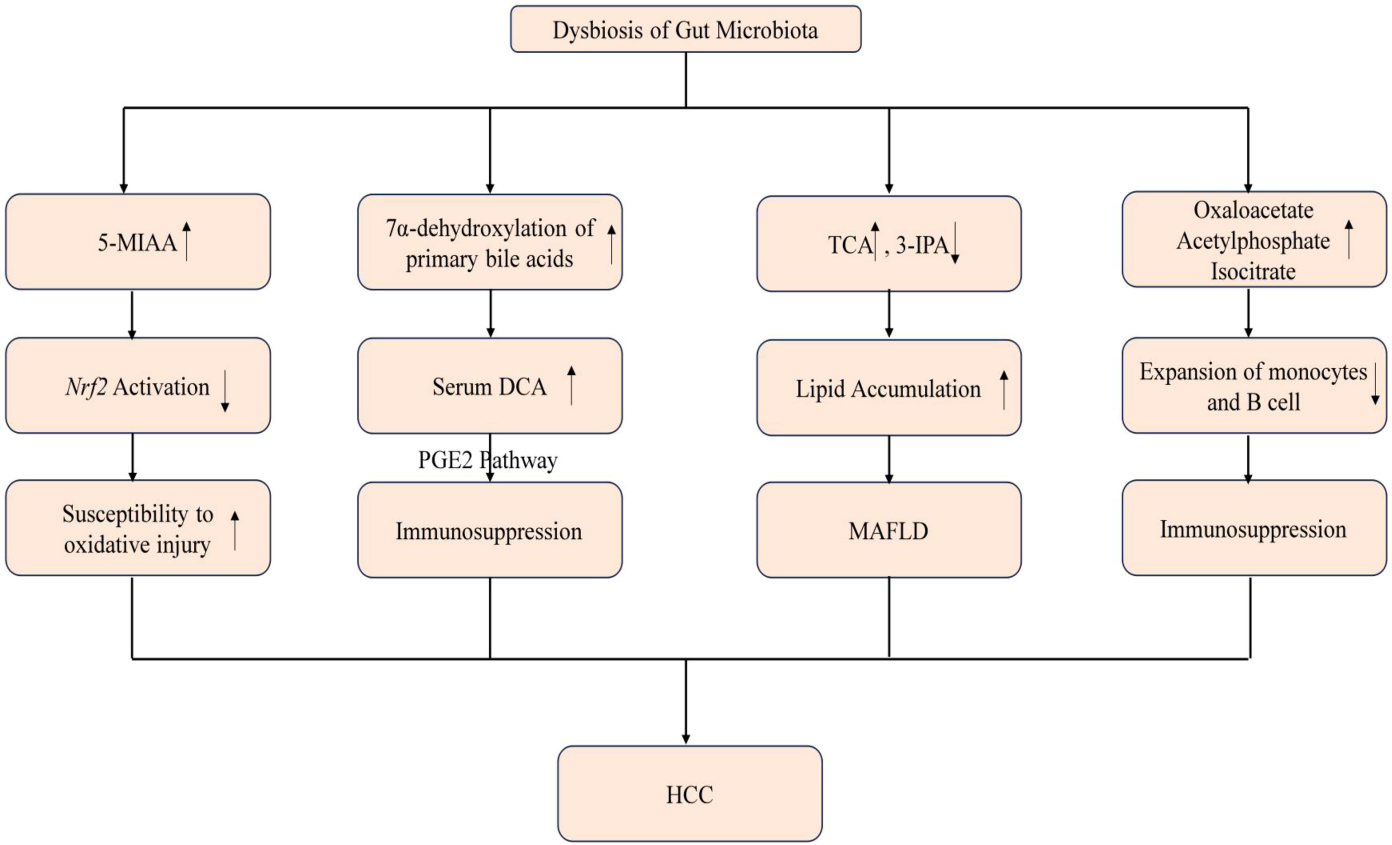
Mechanisms of dysbiosis and the resulting microbial metabolites causing HCC. Imbalances in the gut microbiota, known as dysbiosis, can precipitate alterations in microbial metabolites with significant clinical consequences. An upsurge in 5-MIAA has been linked to diminished activation of the Nrf2 pathway, heightening the risk of oxidative damage to cells. Concurrently, an increase in the 7α-dehydroxylation of primary bile acids leads to higher levels of serum DCA, which is implicated in the suppression of immune functions. Moreover, perturbations in the TCA cycle, along with a reduction in 3-IPA, contribute to lipid accumulation within hepatocytes, setting the stage for MAFLD. Additionally, an elevation in metabolites such as oxaloacetate, acetylphosphate, and isocitrate is associated with a decreased proliferation of monocytes and B cells, possibly compounding the immune dysregulation. Collectively, these biochemical and immunological shifts driven by gut microbiota dysbiosis may culminate in the pathogenesis of HCC. ↑ means increase and the ↓ means decrease.

Previous studies have indicated that dysbiosis can play a role in the development of liver disease and HCC through the influence of bacterial metabolites, potentially in a disease-specific manner (Henao-Mejia et al., 2012; Zheng et al., 2020; Chen et al., 2022; Schneider et al., 2022). In a mouse model of HCC induced by a combination of DMBA (dimethylbenzanthracene) and a high-fat diet, which mimics MASH, there was a significant increase observed in Gram-positive bacterial strains, particularly specific clusters of *Clostridium* (Yoshimoto et al., 2013). This suggests a potential association between these specific bacterial strains and the pathogenesis of MASH-induced HCC (Yoshimoto et al., 2013). Furthermore, this treatment led to elevated levels of DCA in the serum. DCA is a secondary bile acid that is produced through the 7α-dehydroxylation of primary BAs by the bacterial microbiota, particularly the Clostridium clusters. The crucial role of DCA in hepatocarcinogenesis was further demonstrated in experiments where mice exhibited increased HCC development after being supplemented with diets containing DCA. Conversely, inhibiting the 7α-dehydroxylation pathway resulted in reduced HCC formation (Yoshimoto et al., 2013). These findings provide evidence for the involvement of DCA and the bacterial microbiota, specifically the Clostridium clusters, in the development of HCC (Yoshimoto et al., 2013). In collaboration with the TLR2 agonist LCA, DCA facilitated the induction of a senescence-associated secretory phenotype in hepatic stellate cells. This phenomenon subsequently led to the suppression of anti-tumor immunity through prostaglandin E2 (PGE2)-dependent mechanisms. The combined action of DCA and lipoteichoic acid resulted in the promotion of an immunosuppressive microenvironment within the liver, mediated by the secretion of PGE2 (Loo et al., 2017). Collectively, these studies establish a connection between bacterial dysbiosis and altered immune responses mediated by bacterial metabolites and MAMPs. A recent investigation has identified changes in gut bacterial metabolites in MAFLD- HCC, including increased serum levels of taurocholic acid and depleted 3-indolepropionic acid (Zhang et al., 2021). IPA is found to inhibit cholesterol-induced lipid accumulation and cell proliferation, whereas TCA exacerbates cholesterol-induced triglyceride accumulation in a human normal immortalized hepatocyte cell line (LO2) (Zhang et al., 2021). Cholesterol is recognized as a significant lipotoxic molecule contributing to the development of MASH. Additional experiments demonstrated that high dietary cholesterol did not alter the mRNA expression of bile acid synthesis enzymes in the liver. Further *in-vitro* investigations showed that TCA worsened cholesterol-induced triglyceride accumulation in LO2 cells, while IPA suppressed cholesterol-induced lipid accumulation and cell proliferation in MASH–HCC cell lines (HKCI-2 and HKCI-10). All of these indicate that cholesterol promotes the progression of MASH–HCC by modulating host serum metabolites, particularly through increased TCA levels and decreased IPA levels. This evidence suggests that the gut microbiota may influence lipid accumulation in hepatocytes through alterations in metabolites induced by daily dietary intake, thereby contributing to the development of MAFLD-HCC (Zhang et al., 2021). The gut microbiota has been found to produce metabolites that play a protective role against oxidative injury in the liver through various signaling pathways. In a separate study, researchers conducted metabolomic and transcriptional profiling of germ-free mice and conventionalized mice to examine the impact of gut microbiota on liver function. The results revealed an upregulation of the nuclear factor erythroid-derived 2-like 2 (Nrf2) antioxidant and xenobiotic response in animals with a complete microbiome (Saeedi et al., 2020). Furthermore, it was discovered that the human commensal bacterium *Lactobacillus rhamnosus GG* (LGG) strongly activated *Nrf2* in both the liver analog of Drosophila and murine liver (Saeedi et al., 2020). Activation of *Nrf2* was shown to provide protection against oxidative liver injuries induced by acetaminophen overdose and acute ethanol toxicity. Mass spectrometry analysis identified a small molecule activator called 5-methoxyindoleacetic acid (5-MIAA), which is produced by LGG and has been demonstrated to activate hepatic *Nrf2*. This research not only sheds light on the mechanisms by which gut microbiota influence liver health but also provides a novel approach for liver protection against HCC (Saeedi et al., 2020). Recent research has revealed that several metabolites released by dysbiotic gut microbiota play a significant role in the peripheral immune response in metabolism-associated fatty liver disease-related hepatocellular carcinoma (MAFLD-HCC) and can be used as specific markers to identify MAFLD-HCC (Behary et al., 2021). Bacterial extracts obtained from the microbiota of MAFLD-HCC patients have been found to induce a T cell immunosuppressive phenotype characterized by an expansion of regulatory T cells and a reduction in CD8+ T cells (Behary et al., 2021). It suggests that the gut microbiota in MAFLD-HCC is characterized by a distinctive profile in terms of microbiome and metabolomics, which influence the immune response. Three specific metabolites, namely oxaloacetate, acetylphosphate, and isocitrate, have been identified as specific to MAFLD-HCC. Oxaloacetate and acetylphosphate are known intermediates of SCFAs metabolism and were significantly elevated in the feces of MAFLD-HCC subjects compared to those with MAFLD-cirrhosis. Furthermore, bacterial extracts derived from MAFLD-HCC subjects led to the suppression of monocyte and B cell expansion, resulting in alterations in the antigen presenting milieu. The microbiota associated with MAFLD-HCC also induced a cytokine milieu that supports immunosuppression. Notably, the production of pro-inflammatory cytokines IL-2 and IL-12, important for CD8+ T cell expansion and activation, was dampened by the bacterial extracts. On the other hand, IL-6 and IL-10 were induced by MAFLD-HCC bacterial extracts. The presence of Ruminococcus gnavus, Clostridium bolteae, Streptococcus parasanguinis, and Klebsiella pneumoniae, along with reduced abundance of beneficial species such as Faecalibacterium prausnitzii, Alistipes putredinis, and Eubacterium eligens, characterizes MAFLD-cirrhosis (Behary et al., 2021).Additionally, Vibrio parvula and Bacteroides caecimuris were identified as distinguishing factors between MAFLD-HCC and MAFLD-cirrhosis. Similarly, the conversion of tryptophan to indole 3-carboxylate was found to be increased in MAFLD cirrhosis (**Figure 4**).

**Figure 5:**
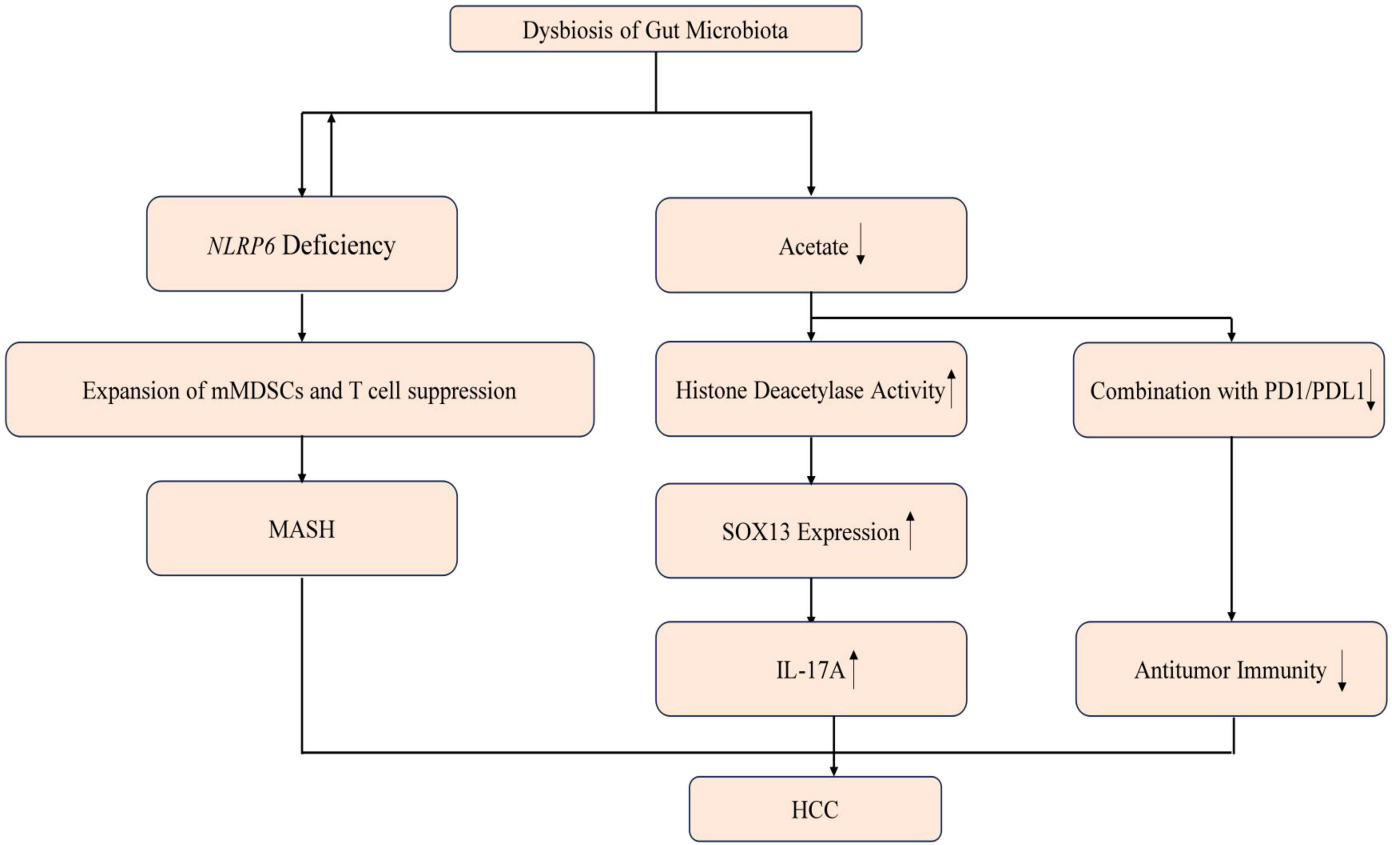
Mechanisms of dysbiosis and the resulting microbial metabolites causing HCC. Dysbiosis can precipitate a shortage of NLRP6, leading to the proliferation of mMDSCs and the suppression of T cells, which may progress to MASH. Additionally, dysbiosis may diminish acetate levels, thereby enhancing histone deacetylase activity and the interaction with the PD1/PDL1 complex. The upregulation of SOX13 expression and IL17, coupled with a reduction in antitumor immunity, can ultimately contribute to the development of HCC. ↑ means increase and the ↓ means decrease.

In some cases, an imbalanced gut microbiota can contribute to the development of a pro-inflammatory environment within the liver (Yu et al., 2010; Dapito et al., 2012). Certain bacterial species or their metabolites may induce the production of inflammatory cytokines and activate immune cells, leading to liver inflammation and injury. On the other hand, a dysbiotic gut microbiota can also negatively impact the inflammatory microenvironment by impairing immune regulatory mechanisms. This can result in a weakened immune response and reduced ability to control inflammation, allowing for the progression of liver diseases (Schneider et al., 2022). An imbalanced or dysbiotic microbiota has been found to induce the expansion of hepatic monocytic myeloid-derived suppressor cells (mMDSC) and suppress T cell abundance in TLR4-dependent manner. This phenotype can be transmitted through fecal microbiota transfer and is reversible upon antibiotic treatment, indicating the high plasticity of the tumor microenvironment. Subsequent research demonstrated that mice lacking the inflammasome sensor molecule NLRP6 develop a dysbiotic colitogenic microbiota composition when housed under SPF conditions. It has been shown that intestinal dysbiosis in *NLRP6*-deficient mice promotes the development of steatohepatitis through the activation of TLR4 and TLR9 pathways. The absence of *NLRP6* exacerbates the progression of liver disease in mice and is associated with intestinal dysbiosis and impaired barrier function. The loss of *NLRP6* influences the hepatic immune environment, while microbiota depletion reshapes the inflammatory microenvironment in the liver and ameliorates steatohepatitis. This study offers a novel approach for the treatment of HCC by targeting the gut-liver axis and modulating the microbiota (Schneider et al., 2022). In mice with HCC, certain species of bacteria, such as *Lactobacillus reuteri*, were found to be significantly reduced in the gut microbiota. This reduction was accompanied by decreased levels of SCFAs, particularly acetate. Transplantation of fecal bacteria from wild-type mice or specifically *L. reuteri* into mice with HCC promoted an anticancer effect, elevated acetate levels, and reduced secretion of IL-17A, a pro-inflammatory cytokine. The mechanism underlying this effect involves acetate reducing the production of IL-17A in hepatic innate lymphoid cells type 3 (ILC3s) by inhibiting histone deacetylase activity. This inhibition leads to increased acetylation of the SRY-box transcription factor *SOX13* at site K30, resulting in decreased expression of *SOX13*. Additionally, the combination of acetate administration with programmed death 1/programmed death ligand 1 (PD-1/PD-L1) blockade significantly enhanced antitumor immunity. Administration of SCFAs, including acetate, in combination with PD-1 monoclonal antibody therapy demonstrated an enhanced antitumor effect in mice with HCC. These findings highlight the potential therapeutic implications of modulating the gut microbiota and SCFA levels, particularly acetate, for enhancing the immune response and improving outcomes in individuals with HCC (Hu et al., 2023) (**Figure 5**).

Gut microbiota influences PLC through microbial metabolites and immune activation. Disruption of the gut barrier and microbial imbalance lead to the release of pathogen-associated molecules and metabolites, which drive liver inflammation, fibrosis, and cancer. Key mechanisms include the activation of TLRs and NLRs, as well as the impact of microbial metabolites like short-chain fatty acids and BAs on liver pathology. The next chapter will examine how targeting the gut microbiome and preserving gut integrity can aid in preventing and detecting HCC at an early stage.

## Making use of the microbiome and gut integrity to prevent HCC and detect at early stage

Currently, the available methods for preventing and treating HCC are limited. However, a thorough review of the mechanisms underlying the gut-liver axis and the collaborative contribution of the gut microbiota to HCC development may provide valuable insights into novel approaches for detecting, preventing, and curing HCC. Recent studies have begun to focus on the specific microbiota profiles in patients with different types of liver diseases, including liver cancers (Grat et al., 2016; Ni et al., 2019; Ponziani et al., 2019; Jia et al., 2021). There is growing evidence supporting the targeting of the microbiome using strategies such as antibiotics, probiotics, fecal microbiota transplantation (FMT), and TLR antagonists (Yu and Schwabe, 2017). These interventions aim to modulate the composition and function of the gut microbiota to potentially prevent or treat HCC. Furthermore, with advancing research on the structure and function of the gut barrier, it is becoming possible to target the integrity of the gut barrier as a preventive measure for HCC. Targeting the microbiome can be instantly effective but also has side effect on patients due to its lack of specificity. As a result, targeting the microbiome for HCC can only be primary prevention in high-risk patients. Many research recently point out the potential use of metabolites of gut microbiota to strengthen the immune responses against the cancer cells or other threats to liver (Loo et al., 2017; Schneider et al., 2022; Hu et al., 2023). We can harness the beneficial metabolites produced by specific gut microbes, such as acetate, to combine with PD1/PDL1 blockade and strengthen immune surveillance and response. At the same time, we can design drugs that specifically eliminate metabolites contributing to immunosuppression. Additionally, besides using the microbiome to treat HCC, the specific profile of the microbiome can be used to identify the stage of HCC, potentially improving patient prognosis.

Strategies that enhance gut barrier function can help protect against the translocation of harmful substances from the gut into the liver, reducing the risk of HCC development. To achieve this, it is important to protect the fragile segments in these barrier structures from damage, thus preventing dysbiosis and the transportation of harmful substances to other organs. For example, the mucus barrier, which is composed of mucus secreted by goblet cells and controlled by the Enteric Nervous System (ENS), plays an important role. The ENS is influenced by neurotransmitters and their corresponding receptors, and any dysfunction in this system can lead to insufficient mucus secretion, facilitating gut microbiota colonization and resulting in primary dysbiosis. Therefore, drugs targeting these neurotransmitters and receptors can be developed to protect the mucus barrier and prevent damage at its earliest stages. Furthermore, a study has shown similarities between the Gut Vascular Barrier (GVB) and the Blood-Brain Barrier (BBB), particularly in the canonical Wnt/β-catenin signaling pathway. An increase in serum levels of PV1 may indicate a loss of gut integrity. If the level of PV1 is confirmed to be associated with HCC, it could potentially be used as a metric for detecting HCC (Brescia and Rescigno, 2021). As for other components of the gut barrier, there are plenty of targets for researchers to study and invent potential drugs to maintain the integrity of gut and probiosis of gut microbiota.

Current methods for HCC prevention and treatment are limited. Research into the gut-liver axis and microbiome suggests that targeting the microbiota with interventions like antibiotics, probiotics, and FMT may offer new approaches. Improving gut barrier integrity could also help prevent HCC. Additionally, utilizing gut microbiota metabolites and profiling the microbiome may enhance HCC detection and treatment. The next chapter will explore future expectations and challenges in HCC research and treatment.

## Outlook for the future: expectation and challenges

Studying the gut-liver axis is a relatively new and promising field with broad applications. However, there are still limitations and challenges that need to be addressed. The gut-liver axis involves interactions among multiple systems, organs, and microbial communities, including the intestines, liver, immune system, and nervous system. This complexity makes it challenging to study and understand the functions and regulatory mechanisms of the gut-liver axis. Additionally, there are variations in the composition and metabolic function of the gut microbiota among different populations, further complicating research efforts.

At present, there is a lack of standardized methods and techniques for studying the gut-liver axis. Different studies use varying experimental animal models, sampling methods, and analysis techniques, which hinders the comparability of results. Therefore, it is necessary to establish more standardized research methods and technical standards to improve the comparison and validation of research findings. Establishing causality between specific changes in the gut microbiota or metabolites and the development of certain diseases remains challenging, despite existing research indicating associations between the gut-liver axis and various diseases. Other factors such as genetics and the environment can also influence disease progression, requiring further research to address these complexities. Although progress has been made in studying the gut-liver axis in animal models and preclinical studies, translating this knowledge into clinical applications poses challenges. The regulatory effects of current treatments and drugs on the gut-liver axis still require widespread validation and confirmation. Furthermore, sample collection, data analysis, and result interpretation in clinical studies demand further efforts. Ethical and safety considerations are crucial in gut-liver axis research, as it involves detecting and modulating the composition and metabolic function of the gut microbiota in human participants. Ensuring ethical compliance and data security during the research process to protect the rights and privacy of participants is of utmost importance. While the study of the gut-liver axis presents exciting prospects, it also faces limitations and challenges. Overcoming these obstacles will lead to a better understanding of the functions and regulatory mechanisms of the gut-liver axis, ultimately facilitating the development of more effective treatment strategies and drugs for related diseases.

### Contemporary clinical trials handling HCC through gut microbiota

Recent clinical trials are exploring the potential of gut microbiota manipulation in treating liver diseases, particularly HCC. The FLORA trial (NCT05690048) assesses the safety and effectiveness of FMT alongside standard immunotherapy to overcome drug resistance in advanced HCC cases. Another study (NCT05170971) investigates the safety and therapeutic effects of FMT on liver failure patients, aiming to optimize the treatment by understanding its impact on the intestinal microecology and the gut-liver axis. Although not directly related to liver cancer, a trial on the treatment of gut graft versus host disease through FMT and dietary fiber supplementation could yield insights relevant to liver health, given the interconnected nature of gut and liver pathologies. These studies signify the burgeoning interest in targeting the gut microbiome as a novel avenue for liver cancer therapy and possibly improving patient outcomes through microbial ecosystem modulation. Most of the clinical trials focus on the FMT methods, in the near future, the researchers will find out way to directly apply the key metabolites of gut microbiota to the human body.

In addition to FMT therapy, SCFA-based treatment presents a promising alternative. Although clinical trials directly investigating SCFA therapy in liver cancer or liver disease patients are limited, its potential remains significant. As previously discussed, SCFAs exhibit diverse therapeutic mechanisms. Acetate and propionate regulate energy metabolism by acting as substrates for fatty acid and cholesterol synthesis and inhibiting hepatic lipogenesis, offering potential benefits for metabolic disorders such as MAFLD. Butyrate and propionate demonstrate anti-inflammatory properties, modulating immune responses via G-protein-coupled receptors and reducing hepatic inflammation, making them promising candidates for liver disease therapy. Additionally, butyrate strengthens gut barrier integrity by enhancing tight junctions, mitigating endotoxemia-related liver damage, and is currently under investigation in cirrhotic patients. However, caution is necessary, as imbalanced SCFA levels, particularly acetate, may contribute to tumorigenesis in certain contexts, underscoring the importance of precise modulation in therapeutic applications. Despite these challenges, SCFAs hold considerable promise as therapeutic agents for liver and systemic diseases, pending further robust clinical validation.

### Defaults and potential improvements to the already clinical treatments

Using microbiome-based therapy for liver cancer requires careful consideration of ethical and safety issues. Privacy and informed consent are critical; participants must provide explicit consent, and their data and identities must be securely protected. Individualized risk assessments are also essential due to microbiome variability, requiring evaluations of potential adverse reactions and expected outcomes. FMT demands rigorous clinical trials to confirm safety, particularly as certain probiotics, like Nissle 1917, have been associated with risks such as colorectal cancer (Jans et al., 2024). Future FMT applications should focus on developing standardized microbial compositions that can be adapted to individual needs, ensuring consistency and safety in treatment protocols.

Proper management of microbial libraries is vital, with strict quality control measures to ensure the safety and viability of microbial strains. Regular monitoring and timely management of adverse effects during treatment are necessary. Adherence to ethical guidelines, collaboration with ethics committees, and the use of robust scientific methods are essential to ensure compliance and reliability throughout the research and treatment process. The scientific community must also further investigate the safety and efficacy of microbiome-based therapies to address these challenges effectively.

Personalized liver cancer treatments rely on tailoring therapies to individual gut microbiome profiles. Advanced sequencing and bioinformatics tools enable the design of customized interventions, such as probiotics and prebiotics, to modulate the microbiome for better outcomes. Continuous monitoring during treatment allows for dynamic adjustments to therapy plans. Clinical validation is crucial to establish the safety and efficacy of these approaches on a broader scale. Harnessing the gut microbiome could optimize liver cancer treatments by improving effectiveness, minimizing side effects, and enabling precise therapeutic strategies, though further research is needed to fully realize this potential.

SCFAs present both opportunities and challenges in clinical applications. Their effects are highly dependent on individual microbiome variability, complicating standardization. SCFAs also exhibit dual roles; while they can suppress inflammation and enhance gut barrier function, they may promote tumorigenesis under certain conditions, necessitating precise dosing and careful monitoring. Additionally, their rapid absorption in the gut reduces bioavailability and limits their therapeutic impact on distant tissues, such as the liver. Innovative delivery systems and sustained-release formulations are required to address these limitations.

Looking forward, advancements in formulation technologies and comprehensive clinical trials will be critical for validating the safety and efficacy of SCFA-based therapies. By overcoming these challenges, SCFAs could play a pivotal role in the treatment of liver diseases, offering enhanced outcomes with reduced systemic side effects.

## Conclusion

This review highlights the critical role of the gut-liver axis in liver disease pathogenesis, particularly HCC. Through detailed analysis, we underscore the importance of microbial metabolites, gut barrier integrity, and systemic immune modulation in driving liver inflammation and carcinogenesis. Furthermore, we address existing controversies in the field, such as the causative relationship between gut dysbiosis and liver disease and the dual role of microbial metabolites like SCFAs, providing a balanced perspective. In the future, longitudinal studies and advanced humanized models are needed to clarify the causal relationships within the gut-liver axis, particularly the roles of specific microbial strains and metabolites in liver disease progression. Developing standardized microbial compositions that can be tailored for individual patient needs will be essential. This includes refining FMT protocols and exploring synthetic microbial consortia with predictable therapeutic outcomes. Bridging the gap between basic research and clinical practice requires robust, multicenter clinical trials to validate the safety and efficacy of microbiota-targeted interventions in diverse patient populations.

By integrating these insights, future efforts can transform the gut-liver axis into a cornerstone of liver disease prevention and treatment, offering hope for personalized and effective therapeutic strategies.
